# Visualization of acetylcholine distribution in central nervous system tissue sections by tandem imaging mass spectrometry

**DOI:** 10.1007/s00216-012-5988-5

**Published:** 2012-04-19

**Authors:** Yuki Sugiura, Nobuhiro Zaima, Mitsutoshi Setou, Seiji Ito, Ikuko Yao

**Affiliations:** 1 Precursory Research for Embryonic Science and Technology (PRESTO), Japan Science and Technology Agency (JST), 7, Gobancho, Chiyodaku, Tokyo 102-0076 Japan; 2 Department of Cell Biology and Anatomy, Hamamatsu University School of Medicine, 1-20-1 Handayama, Higashi-ku, Hamamatsu, Shizuoka 431-3192 Japan; 3 Department of Medical Chemistry, Kansai Medical University, 10-15 Fumizono-cho, Moriguchi, Osaka 570-8506 Japan

**Keywords:** Imaging mass spectrometry, Neurotransmitter, Acetylcholine, MS, MS/MS, Imaging, IMS

## Abstract

Metabolite distribution imaging via imaging mass spectrometry (IMS) is an increasingly utilized tool in the field of neurochemistry. As most previous IMS studies analyzed the relative abundances of larger metabolite species, it is important to expand its application to smaller molecules, such as neurotransmitters. This study aimed to develop an IMS application to visualize neurotransmitter distribution in central nervous system tissue sections. Here, we raise two technical problems that must be resolved to achieve neurotransmitter imaging: (1) the lower concentrations of bioactive molecules, compared with those of membrane lipids, require higher sensitivity and/or signal-to-noise (S/N) ratios in signal detection, and (2) the molecular turnover of the neurotransmitters is rapid; thus, tissue preparation procedures should be performed carefully to minimize postmortem changes. We first evaluated intrinsic sensitivity and matrix interference using Matrix Assisted Laser Desorption/Ionization (MALDI) mass spectrometry (MS) to detect six neurotransmitters and chose acetylcholine (ACh) as a model for study. Next, we examined both single MS imaging and MS/MS imaging for ACh and found that via an ion transition from *m/z* 146 to *m/z* 87 in MS/MS imaging, ACh could be visualized with a high S/N ratio. Furthermore, we found that in situ freezing method of brain samples improved IMS data quality in terms of the number of effective pixels and the image contrast (i.e., the sensitivity and dynamic range). Therefore, by addressing the aforementioned problems, we demonstrated the tissue distribution of ACh, the most suitable molecular specimen for positive ion detection by IMS, to reveal its localization in central nervous system tissues.

## Introduction

Imaging mass spectrometry (IMS), a mass spectrometry (MS)-based molecular imaging technique, is gaining greater popularity as a means of visualizing the distribution of molecular ions in tissue sections and cultured cells [1, 2]. The most characteristic feature of this molecular imaging technique is an MS-based detection principle that has wide versatility, allowing the analysis of many types of analyte molecules, particularly using matrix-assisted laser desorption/ionization (MALDI)-IMS. Consequently, this unique approach provides a novel opportunity to visualize diverse types of molecules directly on tissue surfaces, from small compounds to much heavier biopolymers, which in some cases can be visualized simultaneously [3–5]. MALDI-IMS has been successfully applied for the localization imaging of large structural proteins [6], neuropeptides [7], various types of membrane lipids [8–11], and energy-related secondary metabolites [12] in the brains of healthy and diseased mouse models [13, 14].

This emerging imaging technique was initially developed as a tool for protein imaging [15–17], and most of the early reports on MALDI-IMS described it as a protein or peptide research tool [13, 18, 19]. Conversely, research on detecting and imaging small metabolite molecules has rapidly been expanding [11, 20, 21]. Currently, researchers do not have an established imaging technology for diverse metabolite species, therefore leading to the emergence of IMS as a tool for metabolite imaging. Additionally, the ability of IMS to simultaneously image many types of metabolites is also important because it can be used to visualize the molecular “conversion” of upstream metabolites into downstream metabolites at specific tissue locations in a two-dimensional manner [21].

Despite the promising capabilities of MALDI-IMS, this technique still faces several critical problems regarding its practical use in neurochemical research. One concern is related to its quantitative ability. As the matrix compound and other endogenous isobaric molecules often share nominal mass with the endogenous metabolites of interest, researchers cannot simply interpret the obtained ion intensity as being the analyte concentration. Both peak assignment to specific compounds by mass and careful structural validation by MS/MS or MS/MS imaging are necessary [22]. The other concern is insufficient sensitivity for trace amounts of molecules. This is due to limitations regarding the sample purification process causing a severe ion suppression effect, whereas molecular separation techniques such as gas chromatography, liquid chromatography, and capillary electrophoresis have been traditionally utilized in MS [23].

For this reason, most IMS studies, including those of the author’s group, have reported the analyses of abundant metabolite species, particularly membrane-constituting lipids. However, it is important to expand the application of IMS to much smaller amount of molecules, such as neurotransmitters. The goal of this study was to develop an IMS application to visualize neurotransmitter distribution in the central nervous system (CNS). We raise two technical problems that must be resolved to achieve neurotransmitter imaging: (1) because there are lower concentrations of the bioactive molecules than there are of membrane lipids, we should achieve higher sensitivity and/or selectivity in MS detection, and (2) the molecular turnover of the neurotransmitters is fast; thus, we also should pay attention to the tissue preparation procedure to minimize postmortem changes. To verify these problems, we examined three animal organs using fixation techniques, namely, in situ freezing (ISF) [24] and a conventional fixation with decapitation at different times, and we found that brain samples subjected to ISF exhibited improved IMS data quality in terms of the number of effective pixels and the image contrast, i.e., improved sensitivity and dynamic range.

## Methods

### Materials

Acetylcholine (ACh), γ-aminobutyric acid (GABA), glutamate, dopamine, and serotonin were obtained from Sigma-Aldrich (St. Louis, MO, USA). Norepinephrine was purchased from Wako Pure Chemical Industries Ltd. (Osaka, Japan). α-Cyano-4-hydroxycinnamic acid (CHCA) and 2,5-dihydroxybenzoic acid (DHB) were obtained from Bruker Daltonics (Leipzig, Germany). Male C57BL/6J mice were purchased from SLC (Hamamatsu, Japan).

### Examination of the intrinsic sensitivity using reference standards

To evaluate the efficiency of ionization and interference by the matrix, serially diluted (1.0 × 10^−6^ to 1.0 mg/mL) reference compounds were spotted on a stainless steel plate using DHB and CHCA as matrices. MS detection was performed via laser scanning of the spotted areas on the target plate. Analyte ion intensities and chemical noise from the matrices were calculated from the region of interest (ROI) drawn on each sample spot. The *x*-intercept values were calculated from the intercepts of the semi-logarithmic graphs that were plotted with the log values of the compound concentrations and the signal intensities.

### Sample preparation of tissue sections for IMS analyses

All animals received humane care in accordance with the Japanese Association for Laboratory Animal Science Guidelines, and all of the animal experiments were approved by the Animal Experimentation Committee of Kansai Medical University. For postmortem freezing, mice were deeply anesthetized with pentobarbital to relieve suffering. After decapitation, we removed the brains of the animals within 30 s and the spinal cord within 2 min, and the tissues were frozen in powder dry ice. Frozen tissues were stored at −80 °C until sectioning. For ISF, animals were deeply anesthetized with diethyl ether and the head skin trimmed as described previously [24]. The tip of the head was dipped into liquid nitrogen, with great care taken not to immerse the nose. Frozen brains were dissected with a surgical knife in a refrigerated box at −30 °C. For tissue sectioning and matrix coating for IMS analyses, frozen brains or spinal cords were prepared as 10-μm cryosections on ITO-coated glass slides (Bruker Daltonics) at −20 °C. The sections on the slide glasses were put in a compact desiccator and then brought out, not to be surrounded by frost and not to be degraded by the hydrolytic enzymes. Then, 50 mg/mL DHB in 70 % methanol and 0.1 % trifluoroacetic acid was uniformly sprayed over the samples using a Procon Boy FWA Platinum 0.2-mm caliber airbrush (Mr. Hobby, Tokyo, Japan). The samples were subjected to laser scanning for MALDI-IMS or MS/MS.

### Imaging mass spectrometry

We adopted previously described procedures for MALDI-IMS [20, 25], with some modifications. Chemical compounds and tissue sections were analyzed using a MALDI time-of-flight (TOF)/TOF-type instrument, the Ultraflex II (Bruker Daltonics), and a linear ion trap MALDI LTQ XL™ mass spectrometer (Thermo Fisher Scientific, Waltham, MA, USA). Data were acquired in the positive ion mode using an external calibration method. Calibration compounds which are the aforementioned six neurotransmitters were deposited on the surfaces of the sample support materials to minimize mass shift. MS data by TOF/TOF instrument Ultraflex in the MS mode and 100 laser beam shots were delivered to each data point (Figs. 2 and 3). LTQ XL linear ion trap mass spectrometer was also used for MS measurement (Fig. 4). The intervals between data points were 100 μm (Figs. 4, 5, 6, and 7a, b) and 50 μm (Fig. 7c). MS/MS imaging was performed using an LTQ instrument in the MS/MS mode (Figs. 5 and 6) and Ultraflex in “LIFT” MS/MS mode (Fig. 7). In the MS/MS operation, the data acquisition conditions (i.e., the laser power, collision energy, and number of laser irradiations) were optimized to obtain product ion mass spectra with high signal-to-noise (S/N) ratios for the fragment peaks.

### Image reconstruction

We reconstructed the lipid and neurotransmitter images of interest from the IMS data. Image reconstruction from signals in the spectra was performed using FlexImaging (Bruker Daltonics) and ImageQuest (Thermo Fisher Scientific) software. As the ionization efficiency could vary depending on the matrix–analyte co-crystallization conditions and their sublimation during measurement [19, 25], the absolute intensities of the mass spectra were normalized to the same value of total ion current for the lipid distribution imaging performed by LTQ instrument (Figs. 4, 5, and 6), and this normalization did not apply the normalization to the MS/MS imaging dataset.

## Results and discussion

### Evaluation of the intrinsic sensitivity of six neurotransmitters

**Fig. 1:**
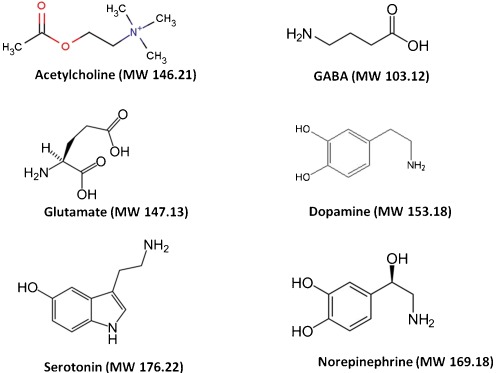
Structure and molecular weights of the six neurotransmitters assessed in this study

**Fig. 2:**
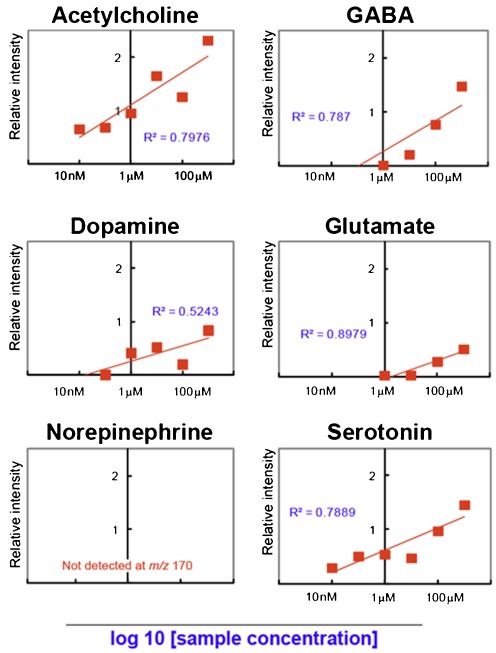
ACh has the highest intrinsic sensitivity among the six assessed neurotransmitters in MALDI-MS. Shown are the intrinsic sensitivity evaluation results of the six neurotransmitters using MALDI-MS with the use of α-cyano-4-hydroxycinnamic acid. The plotted signal intensity values are the difference values between the sample-containing and blank *m*/*z* signal intensities (*Y*-axis, arbitrary units), whereas the *X*-axis is the analyte concentration on a log_10_ scale. The *R* ^2^ values of each collinear approximation are also shown. The values of the *x*-intercept shown in Table 1 represent the detection sensitivity of MALDI-IMS for the six neurotransmitters

In CNS tissues, both specialized structures [26–28] and machinery [29, 30] use neurotransmitters to perform efficient intercellular transduction at chemical synapses, and physiological concentrations of neurotransmitters are much lower than those of typical membrane lipids; neurotransmitter concentrations in brain extracellular fluid are in the low nanomolar range [31–34]. On the other hand, lipids make up half of CNS tissues (by dry weight); therefore, many major lipids exist in concentrations of micromoles lipid per milligram tissue. As a result, lipids are observed as dominant peak clusters in brain MALDI-IMS. [10, 35]. In addition to ion suppression by scan major metabolites, MALDI ionization faces some problems (i.e., interference with the matrix cluster, alterations during sample preparation, and differences in ionization efficiency). Therefore, optimization of typical IMS protocols is necessary to enhance the detection sensitivity for specific compounds—in this case neurotransmitters—in MALDI-IMS. We first evaluated the compound intrinsic sensitivity of six representative neurotransmitters to determine priority for the optimization process (i.e., ease in ionization and detection in the MALDI-IMS experiment), namely, ACh, GABA, glutamate, dopamine, serotonin, and norepinephrine (Fig. 1). For this purpose, we prepared aqueous sample solutions of the transmitters at six different concentrations (tenfold serial dilutions). MS measurements were performed by imaging prepared regions of samples on the target plate, and the analyte ion intensities and the matrix-derived chemical noise were calculated from the ROI drawn on each sample spot. As a result, we found that ACh has the highest intrinsic sensitivity (Fig. 2). The mass spectra from blank matrix spots were acquired; the plotted signal intensity values in Fig. 2 are the difference values between the sample-containing and blank *m/z* signal intensities (*Y*-axis, arbitrary units), whereas the *X*-axis is the analyte concentration on a log_10_ scale. Each plotted square represents the obtained ion intensity at the indicated analyte concentration. We also presented *R* ^2^ values of collinear approximation. Here, the estimated theoretical limit of detection is expected to be in proportion to the *x*-intercept value; therefore, we utilized the value for the intrinsic sensitivity evaluation. For five neurotransmitters, the protonated molecular ions were observed as the major ions, whereas norepinephrine could not be detected as the [M + H]^+^ ion form (data not shown). In Fig. 2, among the six examined neurotransmitters, ACh has the highest *x*-intercept, i.e., it has the highest intrinsic sensitivity. We also examined the different matrix compounds, namely, DHB and CHCA. We performed the same experiment using DHB and compared the *x*-intercept values and matrix interference levels. Table 1 summarizes the results, clearly demonstrating that ACh has the highest intrinsic sensitivity for both CHCA and DHB. Regarding the matrix compound, DHB yielded an approximately two times higher *x*-intercept value in log scale than did CHCA, suggesting that it is a superior matrix for ACh imaging.

**Table 1:** Summary of the detection sensitivity of MALDI-IMS for the six neurotransmitters using DHB and CHCA as matrices

	DHB		CHCA	
	*x*-intercept (nM)	Matrix interference	*x*-intercept (μM)	Matrix interference
Acetylcholine	0.250	+	0.350	+
Serotonin	1.032	++	1.000	−
Dopamine	18.70	++	8.110	−
GABA	87.24	−	6.865	−
Glutamate	1934	−	73.40	+
Norepinephrine	N.D.	−	N.D.	−

### MS/MS ion transition improved the S/N ratio for detecting ACh-derived signals

**Fig. 3:**
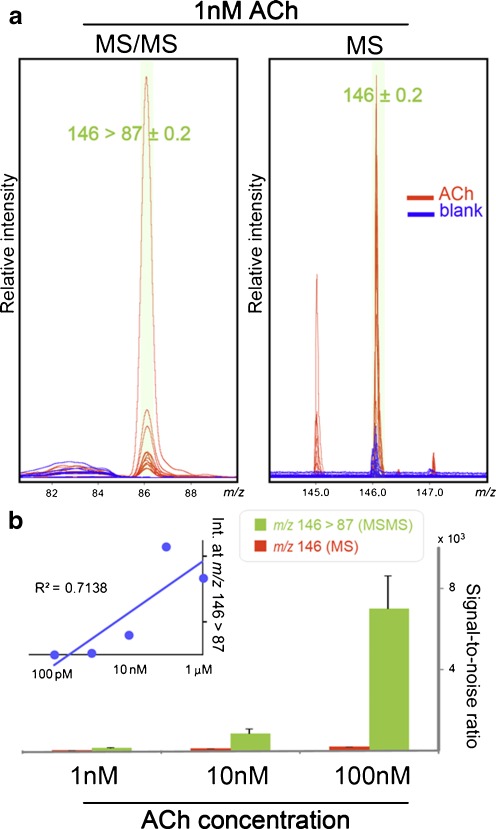
MS/MS ion transition from *m/z* 146 > 87 improved the S/N ratio for detecting the ACh-derived signals. **a** Detection of 1 nM ACh by MS/MS measurement via an ion transition from *m/z* 146 to 87 (*left*) and by a single MS measurement at *m/z* 146 (*right*). For both, although the spectra obtained from the sample-containing spots (*red*) displayed clear ACh-derived signals, those from blank DHB spots (*blue*) only exhibited matrix-derived background peaks; however, in the single MS measurement, a DHB-derived peak observed around *m/z* 146 interfered with the detection of ACh in the MS mode. Both measurements were repeated for approx. 15 shots, and the obtained spectra were merged. **b** Bar chart presenting the S/N ratios calculated by dividing the signal intensity at *m/z* 146 > 87 (MS/MS) and *m/z* 146 (MS) by the corresponding background peak intensity obtained from blank spots. *Inset* shows the peak intensity plot at *m/z* 146 > 87 (*Y*-axis) along with the dilution series of the ACh standard sample (*X*-axis). Data were acquired by the TOF/TOF instrument in the MS or LIFT mode

Furthermore, monitoring ion transition by MS/MS measurement improved the S/N ratio of the ACh-derived signal by eliminating matrix interference. Previous studies reported that collision-induced dissociation of ACh yields a major fragment ion at *m/z* 87 arising from the loss of trimethylamine [36]. Therefore, we compared the S/N ratio and sensitivity of ACh-derived signals between single MS measurements (ion at *m/z* 146) and MS/MS measurements (ion at *m/z* 146 > 87). Figure 3a shows the results of 1 nM ACh standard measurements by MS/MS (left) and single MS measurements (right). For both measurements, the spectra obtained from the sample-containing spots (red) exhibited clear ACh-derived signals, whereas those from blank DHB spots (blue) only exhibited matrix-derived background peaks. In particular, in the single MS measurement, a DHB-derived peak observed at *m/z* 146 interfered with the detection of ACh in the MS mode. Conversely, there was no observable matrix interference at *m/z* 87 in the product ion spectrum of *m/z* 146. As expected, this advantage offered an excellent S/N ratio that is superior to that of the single MS detection, even for the measurement of a trace sample concentration. Figure 3b presents a bar chart showing the S/N ratio calculated by dividing the signal intensity at *m/z* 146 > 87 (MS/MS) and *m/z* 146 (MS) by the corresponding background peak intensity obtained from the blank spots. These MS/MS measurements provided an S/N ratio exceeding 100 for the ion transition signal even at a sample concentration of 1 nM, owing to the low background peak intensity. We also note that the absolute intensity of *m/z* 146 > 87 was not drastically improved, and therefore, the lower detection limit of ACh was almost equal to that of the single MS measurement (Fig. 3b, inset).

### Distribution imaging of ACh in mouse spinal cord sections

**Fig. 4:**
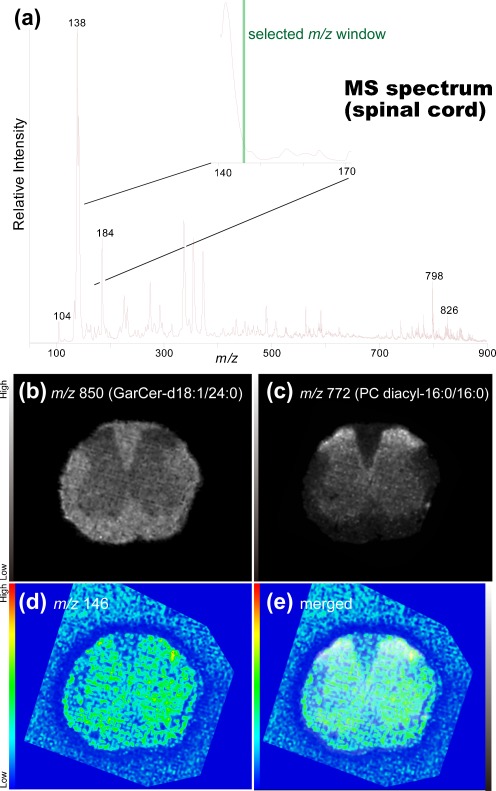
Single-stage mass spectrometry imaging was not useful for ACh distribution imaging in the mouse spinal cord section. **a** Averaged mass spectrum of all spectra obtained by imaging mass spectrometry of the spinal cord section. The expanded spectrum (*inset*) shows that the intense ion peak at *m/z* 141 hid the mass window for the ACh-derived ion peak that could be observed at *m/z* 146. **b**–**d** Reconstructed ion intensity maps for ions at *m/z* 850 that were derived from galactosylceramide (*GalCer*) (**b**) and ions at *m/z* 772 derived from the phosphatidylcholine PC (*diacyl-16:0/16:0*) (**c**). In addition, an ion image at *m/z* 146 was visualized (**d**), although merging of that image with GalCer (**e**) reveals that this ion was distributed both inside and outside the tissue sections, suggesting that it contained matrix-derived chemical noise. Data were acquired by the LTQ instrument with single MS measurements

Having demonstrated that ACh has the highest intrinsic sensitivity among the analyzed major neurotransmitters, we proceeded to perform imaging experiments of the distribution of ACh using MALDI-IMS. Here, we utilized mouse spinal cord sections as a first examination sample because the distribution pattern of the cholinergic neuron in the spinal cord is simple and well described [37]. The white matter outside the spinal cord is composed of nerve fibers, whereas the inside gray matter mainly consists of neuronal cell bodies. Therefore, we could easily evaluate the appropriateness of the obtained imaging results by comparing the ion images and anatomical features of the same section. The initial experiment was performed using single-stage MS imaging (Fig. 4). However, as can be seen in panel (a), we failed to detect an isolated signal peak for ACh at *m/z* 146 due to a rather intense peak at *m/z* 141, the foot of which covered the mass range containing the ACh signal at *m/z* 146. The existence of this interference peak resulted in a non-tissue-specific ion distribution at *m/z* 146, suggesting that this peak did not represent ACh localization (Fig. 4d).

Conversely, peak clusters derived from phospholipids were clearly observed at a mass range of 700 < *m/z* < 900 in the same section, and their characteristic distribution patterns were useful for understanding the anatomical features of the tissue sections. For example, because the glycolipid galactosylceramide counts myelin sheaths as one of its major lipid components, the ion at *m/z* 850 derived from galactosylceramide was highly localized in the white matter region of the spinal cord. In addition, a phosphatidylcholine (PC) molecular species observed at *m*/*z* 772 [10, 38], PC (diacyl-16:0/16:0, K^+^ adduct), was localized in the gray matter region at *m/z* 772, especially in the dorsal spinal horn.

### Tandem MS imaging of ACh in mouse spinal cord sections

**Fig. 5:**
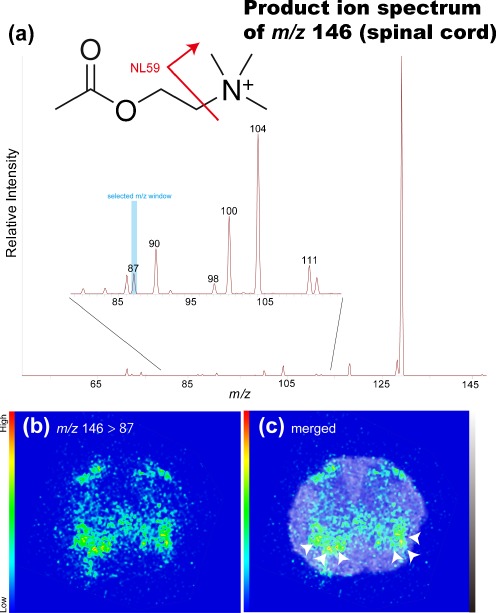
Tandem IMS was useful for determining the distribution of ACh in mouse spinal cord sections. **a** The averaged product mass spectrum of all spectra obtained by IMS of spinal cord sections. The expanded spectrum (*inset*) shows that the detection of the ACh-derived product ion at *m/z* 87 was derived from the neutral loss of the trimethylamine group (*NL59*) from the intact ion (see the presented molecular structure). **b** Reconstructed ion intensity maps for the ion transition from *m/z* 146 to *m/z* 87 (tandem MS signature of ACh) and its merged image on the galactosylceramide ion distribution map. **c** ACh was localized in the region containing cholinergic motor neurons (*arrowheads*). Data were acquired by the LTQ instrument in the MS/MS mode

We further proceeded to MS/MS imaging selection of ions at *m/z* 146 as a precursor; this method was in turn revealed to produce high S/N ratios (Fig. 5). In these experiments, we detected a product ion peak at *m/z* 87, the characteristic and most intense fragment ion of the ACh molecule [39] which was derived from the neutral loss of the trimethylamine group (NL59) from the intact ion (panel a). Figure 3b shows the distribution of this product ion at *m/z* 87 (i.e., ion transition from *m/z* 146 to *m/z* 87), revealing that it is clearly localized in specific regions of the spinal cord section. We obtained the lipid signals on the same tissue section which was used for ACh imaging. The merged image containing the ion image at *m/z* 772 (Fig. 5c) reveals the intense localization of ACh in the region containing rich motor neurons in the anterior horn [40, 41], and the overall distribution pattern was found mainly in the gray matter region.

We noticed high-intensity ACh signals in both the ventral and dorsal horns (Fig. 5c). ACh is an important modulator of motor and sensory processing, especially at the spinal level, at which point pain-related nociceptive stimuli enter the CNS, which integrates the stimuli and relays them to the brain. There are large cholinergic motor neurons in the spinal anterior horn [40]; thus, it is reasonable that ACh was abundantly detected in ACh-producing cells containing choline acetyltransferase (ChAT), the ACh-synthesizing enzyme [42]. The source of ACh in the sensory spinal cord has not been clearly established; however, the presence of a dense plexus of cholinergic fibers has been reported in the spinal lamina II [43] and III [44] with the use of anti-ChAT immunolabeling. In addition to immunohistochemical findings, exogenous ChAT was found in the ventral and dorsal horns in ChAT promoter-driven EGFP-expressing mice [45].

We directly detected the localization of ACh using IMS, whereas conventional methods such as immunohistochemistry indirectly described the localization of ACh sites of action with its receptors, transporters, and synthetic or catabolic enzymes. ISH data of the mouse spinal cord from the Allen Brain Atlas (http://www.brain-map.org/) demonstrate that the distribution of IMS images is reasonable compared with the localization of mRNA for ACh-related molecules such as ChAT, acetylcholinesterase (AChE), nicotinic receptor (nAChR), muscarinic receptor (mAChR), vesicular acetylcholine transporter (VAChT), and choline transporter (ChT). Many previous results matching our IMS data also match those of ISH and/or immunohistochemistry for ACh-related molecules including ChAT [42, 44, 46], AChE [47], nAChR [48], and VAChT [46, 49, 50]. Through its action on spinal cholinergic receptors, endogenous ACh participates not only in motor action but also in the setting of nociceptive thresholds and in the effect of clinically relevant analgesics; thus, the direct detection of ACh in the spinal cord will be useful for assessments such as evaluations of therapeutic strategies.

### Distribution imaging of ACh in mouse sagittal brain sections

**Fig. 6:**
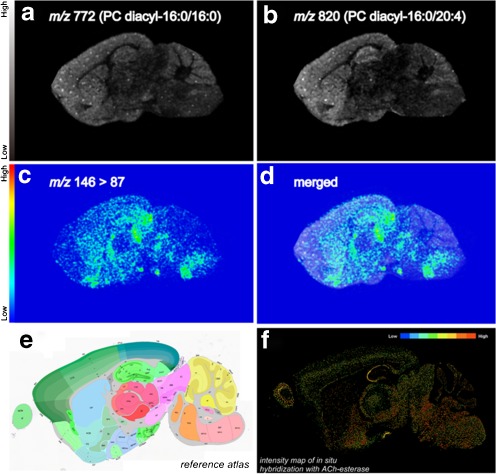
Tandem imaging mass spectrometry was useful for ACh distribution imaging in mouse brain sections. **a**, **b** Reconstructed ion intensity maps for ions at *m/z* 772 derived from PC (*diacyl-16:0/16:0*) (**a**) and ions at *m/z* 820 derived from PC (*diacyl-16:0/20:4*) (**b**). **c** Reconstructed ion intensity maps for the ion transition from *m/z* 146 to *m/z* 87 (the tandem mass spectrometry signature of ACh) and its merged image with the ion distribution map of PC (diacyl-16:0/16:0) (**d**). **e** Scheme of the mouse brain map from the Allen Brain Atlas (http://www.brain-map.org/). **f** Image of the intensity map of in situ hybridization with AChE from the Allen Brain Atlas showing that ACh was localized in the AChE-abundant region. Data were acquired by the LTQ instrument in the MS/MS mode

Having demonstrated that ion transition imaging from *m/z* 146 to *m/z* 87 is an effective IMS protocol for determining the distribution of ACh in spinal cord sections, we applied the technique to study sagittal mouse brain sections. We successfully obtained an ACh distribution image (Fig. 6). The ion transition signal at *m/z* 87 was abundant in the postsubicular-retrohippocampal region, superior colliculus, medial fenuculate complex, subparafascicular nucleus, peripeduncular nucleus, lateral dorsal nucleus of thalamus, reticular nucleus of the thalamus, fundus of the striatum, olfactory tubercle, reticular part of the substantia nigra, middle cerebellar peduncle, parvicellular reticular nucleus, facial motor nucleus, medial vestibular nucleus, parabrachial nucleus, and pontine reticular nucleus (Fig. 6). The distribution of ACh in the mouse brain reconstructed from IMS data better fits the patterns of ACh flow areas than that of ACh-producing areas. For example, the ion transition signal at *m*/*z* 87 was abundant in the superior colliculus and nucleus of the thrums and was observed in the hippocampus and cerebral cortex in a diffused manner in cholinergic innervation sites [51]. In contrast, the nuclei of ACh-generating neurons produced a small signal in areas such as the nucleus basalis of Meynert and the medial septal nucleus. Based on the Allen Brain Atlas ISH AChE data (Fig. 6e, f) and previous reports, ACh was mainly distributed at the neurotransmitter release sites rather than at the nuclei [52, 53].

### The ISF procedure protected endogenous ACh from postmortem degradation, resulting in improved MS/MS imaging sensitivity

Earlier studies described that the molecular turnover of ACh is rapid [54], and therefore, we should pay attention to the tissue preparation procedure to minimize postmortem changes. In this context, optimization of the organ sampling protocol has remained a critical issue because major degradation of the various metabolites could occur in tissues even within a second after respiratory arrest, especially concerning small molecules involved in signal transduction. As described below, we finally evaluated the extent of ACh postmortem changes among brain samples that were subjected to ISF, which ensures adequate perfusion until the arrival of the freezing front to avoid unnecessary autolysis, and postmortem freezing (decapitation before freezing). As a result, we found that ISF was the best method for ACh imaging.

**Fig. 7:**
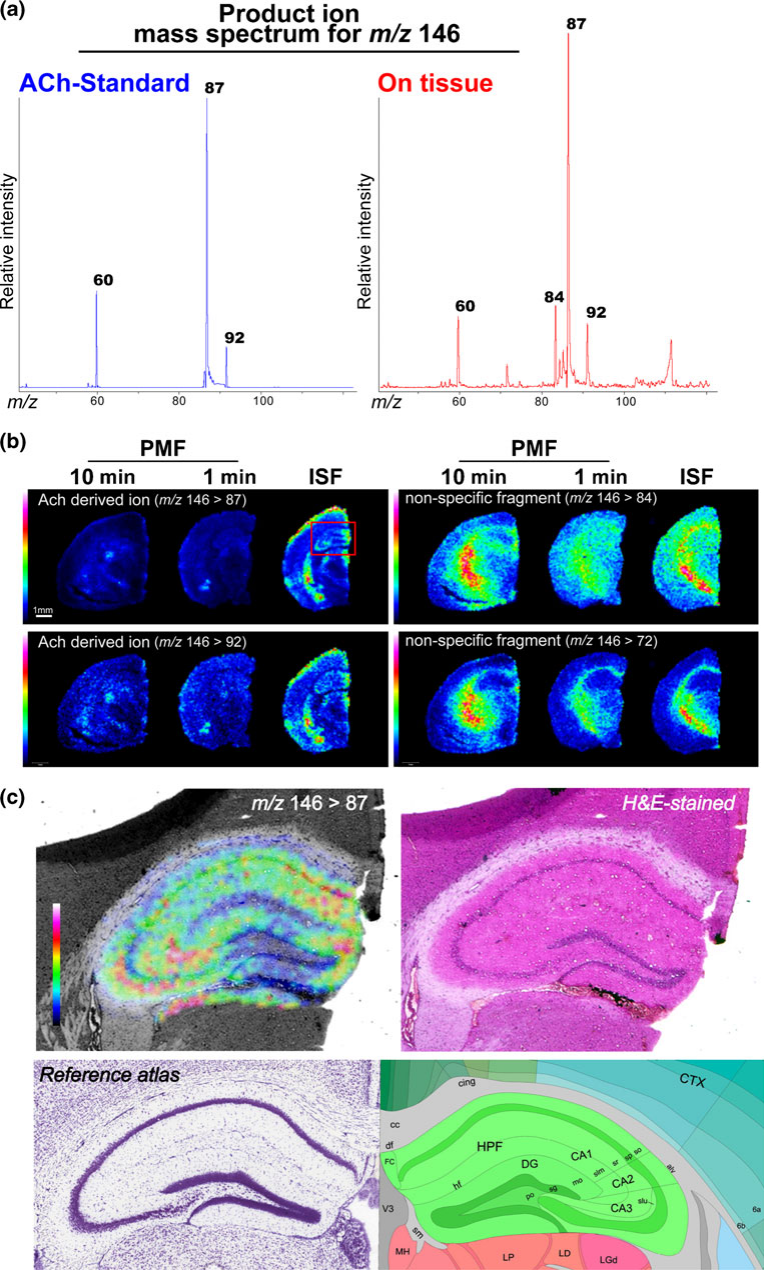
ISF procedure protected endogenous ACh from postmortem degradation, resulting in improved MS/MS imaging sensitivity. **a** Averaged product ion mass spectrum of *m/z* 146 obtained from the ACh standard (*left*) and on brain tissue (*right*). **b** Representative ACh and other ion distribution images visualized by MS/MS imaging for *m/z* 146, obtained from three groups of mice treated with different animal fixation methods; the tissue fixation techniques were in situ freezing (*right*) and postmortem freezing with decapitation, in which brain extraction was performed within 1 min (*center*) and 10 min (*left*) of decapitation. Imaging results of the two major ACh-derived ion transition signatures, *m/z* 146 > 87 and *m/z* 146 > 92, exhibited similar distribution patterns, indicating that the correct distribution map for ACh was obtained. Conversely, the other fragment ions (84 and 72) exhibited a white matter-specific ion distribution. Obviously, the ISF sample provided the most sensitive ACh signal. **c** Expanded ACh distribution image of the hippocampus of an ISF-treated brain (*left*) and an optical image of the same brain section stained with H&E (*right*). For reference, corresponding brain atlas images are also shown (*bottom*). Data were acquired by the TOF/TOF instrument in the MS or LIFT mode

Figure 7a shows the averaged product ion mass spectrum of *m*/*z* 146 obtained from the ACh standard (left) and on the brain tissue (right). The imaging results of the two major ACh-derived ion transition signatures, *m/z* 146 > 87 and *m/z* 146 > 92, exhibited similar distribution patterns, indicating that the correct distribution imaging for ACh was achieved. Conversely, the other fragment ions exhibited a white matter-specific ion distribution.

Having observed the ACh-specific ion transitions of the brain section, distribution maps of the major fragment ions were then reconstructed and evaluated (Fig. 7b); the quality of these ACh-derived ion images was assessed among three groups of mice that were treated with different animal fixation methods, namely, ISF (right) and postmortem freezing with decapitation in which brain extraction was performed within 1 min (center) and 10 min of decapitation (left). As shown in the figure, the IMS data quality in terms of the number of effective pixels and the image contrast, i.e., sensitivity and dynamic range, was drastically improved in the ISF brain sample compared with that in the postmortem freezing-treated group, clearly demonstrating that the proper sample preparation technique is necessary for correct ACh imaging. Furthermore, increased sensitivity enables the distribution mapping of ACh at a higher spatial resolution. Figure 7c shows the expanded ACh distribution image of the hippocampus of ISF-treated brains, revealing a unique ACh distribution pattern even within hippocampal substructures.

## Conclusion

Among the neurotransmitters, ACh was most sensitively detected using the positive ion mode of MALDI-MS. Based on this fundamental study, we successfully reconstructed ACh distribution images in the mouse brain and spinal cord from the scan data obtained from MS/MS ion transitions. Furthermore, the IMS data quality in terms of the number of effective pixels and the image contrast, i.e., the sensitivity and dynamic range, was drastically improved in ISF-treated brain samples, clearly demonstrating that the ISF sample preparation technique is necessary for precise ACh imaging.

The localization of ACh obtained in this study coincided agreeably with the expected localization based on the known distribution of the ACh-degrading enzyme, especially in the spinal cord and part of the brain. Therefore, we conclude that MS/MS-based IMS could be useful for neurotransmitter imaging and can be practically used in the field of neuroscience.

## Abbreviations

ACh: Acetylcholine
CE: Capillary electrophoresis
ChAT: Acetyltransferase
CHCA: α-Cyano-4-hydroxycinnamic acid
CNS: Central nervous system
DHB: 2,5-Dihydroxybenzoic acid
GABA: Gamma-aminobutyric acid
GC: Gas chromatography
IMS: Imaging mass spectrometry
ITO: Indium tin oxide
LOD: Limit of detection
LC: Liquid chromatography
MALDI: Matrix-assisted laser desorption/ionization
ROI: Region of interest
TLC: Total ion current
